# Chondroitin Sulfate-Based Self-Assembling Nanoprodrug for Controlled Methotrexate Delivery in Cancer Therapy

**DOI:** 10.3390/molecules31152578

**Published:** 2026-07-24

**Authors:** Ludovica Scorzafave, Michele Pellegrino, Giuseppe Cirillo, Marco Fiore, Roberta Pino, Diana Amantea, Antonella Leggio, Fiore Pasquale Nicoletta, Francesca Iemma, Manuela Curcio

**Affiliations:** Department of Pharmacy Health and Nutritional Science, University of Calabria, 87036 Rende, Italy; ludovica.scorzafave@unical.it (L.S.); michele.pellegrino@unical.it (M.P.); marco.fiore@unical.it (M.F.); roberta.pino@unical.it (R.P.); diana.amantea@unical.it (D.A.); antonella.leggio@unical.it (A.L.); fiore.nicoletta@unical.it (F.P.N.); francesca.iemma@unical.it (F.I.); manuela.curcio@unical.it (M.C.)

**Keywords:** chondroitin sulfate, self-assembling nanoparticles, methotrexate, pH-responsive polymeric prodrug, cancer therapy

## Abstract

In this study, a pH-responsive chondroitin sulfate–methotrexate (MTX) polymeric prodrug was synthesized through Schiff base formation between oxidized chondroitin sulfate and MTX. The resulting amphiphilic conjugate exhibited a conjugation degree of 184 mg MTX per g conjugate and spontaneously self-assembled into stable nanoparticles (CSMXPs) with a mean diameter of 120 ± 10 nm, a polydispersity index of 0.24, and a critical aggregation concentration of 4.7 × 10^−4^ mg mL^−1^. Drug release studies demonstrated a marked pH-dependent behavior, with complete MTX release after 24 h at pH 5.0 and a sustained release profile under physiological conditions. The release mechanism followed reversible first-order kinetics and was accelerated by acid-catalyzed hydrolysis of the imine linkage. Biological evaluation revealed enhanced therapeutic selectivity of CSMXPs compared with free MTX. At 36 μM MTX-equivalent concentration, CSMXPs reduced HeLa cell viability to 37%, while maintaining MCF-10A viability above 88%, whereas free MTX decreased viability in both cell lines (51% and 65%, respectively). Fluorescence confocal microscopy confirmed efficient nanoparticle uptake by cancer cells. These findings demonstrate that CSMXPs represent a promising self-assembling nanoprodrug platform for selective and targeted cancer therapy.

## 1. Introduction

Prodrugs are inactive, bioreversible compounds rationally designed to undergo in vivo conversion into their pharmacologically active forms after administration [1,2,3]. Over recent decades, the prodrug approach has become a powerful strategy to enhance drug bioavailability and therapeutic efficacy by overcoming key biopharmaceutical, pharmacokinetic, and pharmacodynamic limitations [4,5]. This strategy effectively addresses major drawbacks of conventional therapeutics, including poor chemical stability and water solubility, limited permeability across biological barriers, inadequate target selectivity, rapid metabolism, systemic toxicity, and poor patient compliance [6].

Over recent decades, advances in polymer science have led to the development of site-specific drug delivery systems based on polymer–drug conjugates. The covalent linkage of a therapeutic agent to a polymeric carrier generates a polymeric prodrug, an inactive precursor capable of circulating in the bloodstream and releasing the active drug only upon exposure to specific biological stimuli. Polymeric prodrugs offer several pharmacological advantages, including prolonged circulation time, controlled drug release, improved stability, and the ability to exploit the enhanced permeability and retention (EPR) effect for passive tumor targeting. Furthermore, conjugation to macromolecular carriers can reduce renal clearance and immunogenicity, thereby enhancing drug accumulation at the target site while minimizing systemic exposure [7,8].

Among the various materials investigated as polymeric carriers, polysaccharides have attracted considerable attention owing to their biocompatibility, biodegradability, cost-effectiveness, and therapeutic synergy. Their abundance of reactive functional groups, including hydroxyl, carboxyl, and amino moieties, enables straightforward chemical modification and drug conjugation [9,10,11]. Among these biopolymers, chondroitin sulfate (CS) is particularly attractive due to its non-toxic and non-immunogenic nature. CS is an anionic sulfated glycosaminoglycan widely distributed in vertebrates, invertebrates, and bacterial species. Its favorable biological properties have promoted its extensive use in the design of advanced nanocarriers for chemotherapeutic and theranostic applications [12,13].

Beyond its favorable safety profile, CS has gained increasing attention as a platform for cancer-targeted drug delivery. The ability of sulfated glycosaminoglycans to interact with cellular receptors and extracellular matrix components associated with tumor development, including CD44, scavenger receptors, and cell-surface proteoglycans, may promote the preferential accumulation and uptake of CS-based nanocarriers in malignant tissues [14,15]. These properties, together with its biodegradability and versatile chemical functionality, make CS a particularly attractive candidate for the design of advanced anticancer delivery systems [16,17].

For successful incorporation into a polymeric prodrug platform, a drug candidate should possess high pharmacological potency, suitable reactive functional groups for covalent conjugation, and sufficient stability within the macromolecular construct to prevent premature release or elimination [18]. Methotrexate (MTX), a folate analog antimetabolite widely used in cancer therapy since the 1950s, fulfills these requirements. MTX exerts its antitumor activity by inhibiting DNA synthesis and cell proliferation. However, despite its clinical relevance, MTX suffers from several limitations, including poor aqueous solubility, limited membrane permeability, short biological half-life, inadequate tumor accumulation, and unfavorable tissue distribution [19,20]. Consequently, increasing the administered dose often leads to severe systemic toxicity without a proportional improvement in therapeutic efficacy.

Several polymeric MTX prodrugs have been reported in the literature, employing carriers such as poly(ethylene glycol), N-(2-hydroxypropyl)methacrylamide (HPMA) copolymers, dextran, and chitosan to improve the pharmacokinetic profile and therapeutic performance of the drug [21,22,23,24]. Although these systems have demonstrated enhanced circulation time and reduced systemic toxicity, many rely on complex synthetic procedures or lack efficient stimuli-responsive release mechanisms capable of selectively triggering drug liberation at the tumor site. In this context, polysaccharide-based prodrugs incorporating environmentally sensitive linkages represent an attractive alternative. Nevertheless, the development of CS-based MTX conjugates remains relatively unexplored, particularly those designed to combine nanoparticle self-assembly with pH-responsive drug release [25].

In the present work, an oxidized chondroitin sulfate–methotrexate (CS_ox_MTX) prodrug was synthesized through the formation of pH-sensitive imine linkages between the polysaccharide backbone and the anticancer drug. The resulting amphiphilic conjugate was induced to self-assemble into nanoparticles by the solvent displacement method, yielding a stable nanosized prodrug system (CSMXPs) in which the carrier, the therapeutic agent, and the release-triggering moiety are integrated into a single platform. The acid-labile imine linkage was specifically selected to promote MTX release within the intracellular endosomal/lysosomal compartments through hydrolytic cleavage, enabling controlled and stimuli-responsive drug delivery. The synthesized nanoprodrug was subsequently characterized in terms of its physicochemical properties, pH-responsive drug release, and in vitro anticancer activity. The combination of nanoparticle self-assembly, pH-triggered release, and the favorable biological properties of chondroitin sulfate is expected to enhance therapeutic selectivity while reducing systemic toxicity.

## 2. Results and Discussion

### 2.1. Synthesis and Characterization of Methotrexate Prodrug

**Figure 1:**
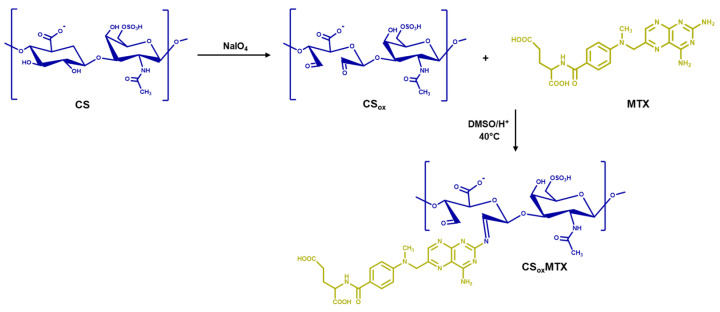
Schematic representation of two-step synthetic procedure: CS oxidation and MTX coupling by Schiff base formation.

The methotrexate prodrug was synthesized through a two-step procedure involving the preliminary oxidation of chondroitin sulfate (CS) followed by the conjugation of methotrexate (MTX) to the resulting oxidized polysaccharide. Literature reports suggest that both aldehyde groups of CS_ox_ are susceptible to nucleophilic attack, although their relative reactivities are difficult to establish [26,27]; therefore, Schiff base formation is likely to occur through aldehyde functionalities. Moreover, both amino groups of MTX can potentially participate in the condensation reaction; however, the amino group at position 2 of the pteridine ring is considered more nucleophilic and is thus expected to react preferentially with the aldehyde functionalities of CS_ox_ [28]. Accordingly, the proposed structure of the CS_ox_MTX conjugate is depicted in Figure 1.

CS oxidation was carried out using sodium periodate (NaIO_4_) [29], which selectively cleaves vicinal hydroxyl groups, generating dialdehyde functionalities along the polysaccharide backbone that serve as reactive sites for subsequent conjugation with nucleophilic species [30].

The oxidation degree (OxD%) of CS_ox_ (27%), expressed as the molar ratio of aldehyde groups to the original vicinal hydroxyl groups of CS, was quantified using the hydroxylamine hydrochloride titration method. Aldehyde groups react with NH_2_OH·HCl to produce stable oxime derivatives, releasing an equivalent amount of hydrochloric acid. The liberated acid was then quantified by titration in the presence of methyl orange as an indicator, allowing the aldehyde content and, consequently, the oxidation degree of the sample to be calculated according to Equation (1).

(1) $$OxD\%=\frac{{(Vol}_{NaOH}\times N_{NaOH})/2}{m_{CS}\;/\;{MW}_{CS-RU}}\times 100=\frac{mol\;of\;CHO}{mol\;of\;CS–RU}\times 100$$

where *CS–RU* represents the *CS* repeating unit.

Previous studies have shown that increasing the oxidation degree enhances the number of aldehyde groups available for further derivatization, thereby improving conjugation efficiency [31]. However, excessive oxidation may compromise biocompatibility because residual aldehyde groups can interact with cellular proteins and promote oxidative stress [32,33]. Therefore, intermediate oxidation conditions were selected to achieve a balance between functionalization efficiency and biological safety.

**Figure 2:**
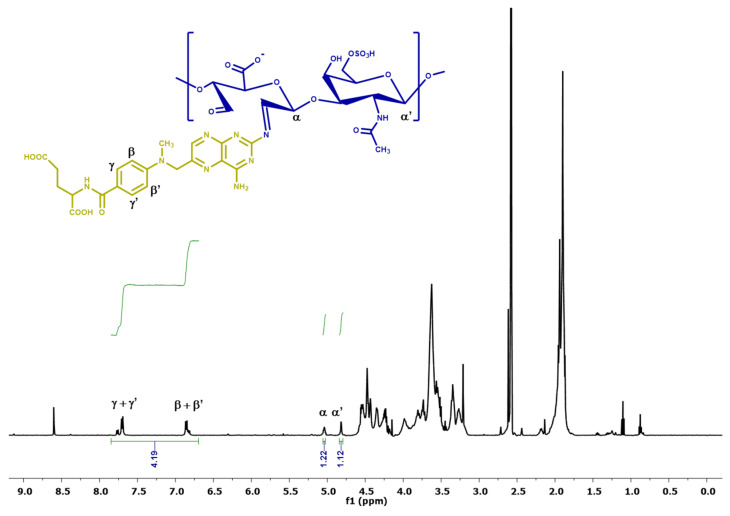
^1^H-NMR spectrum of CS_ox_MTX in DMSO-d6/D_2_O.

Subsequently, CS_ox_ was reacted with MTX under mildly acidic conditions leading to the formation of Schiff base linkages (CS_ox_MTX) between the aldehyde groups of the polysaccharide and the amino groups of the MTX pteridine moiety. Successful conjugation was confirmed by ^1^H-NMR spectroscopy (Figure 2).

Characteristic MTX resonances were clearly detected at 6.85 ppm and 7.69 ppm, corresponding to the four benzoyl aromatic protons, together with the signal at 8.60 ppm attributed to the aromatic proton of the 2,4-diamino-6-pteridinyl moiety [34], while the signals at 4.82 and 5.03 ppm were assigned to the CS_ox_ anomeric protons [35]. Comparison of the integrated area of the MTX aromatic proton signals with that of the α and α’ anomeric proton signals of oxidized CS, which were absent in the ^1^H-NMR spectrum of pristine CS (Supplementary Figure S1), allowed the determination of the conjugation degree (CD%), expressed as moles of MTX per mol of CS_ox_ repeating units according to the following Equation (2).

(2) $$CD\%=\frac{I_{{MTX}^{m}}/N_{{MTX}^{m}}}{I_{{CS}_{ox}^{m}}/N_{{CS}_{ox}^{m}}\;}\times 100\;$$

where $I_{{MTX}^{m}}$ and $N_{{MTX}^{m}}$ represent the integrated area and the number of the aromatic protons (β, β’, γ, γ’) signals of MTX moieties in the CS_ox_MTX conjugate, respectively, whereas $I_{{CS}_{ox}^{m}}$ and $N_{{CS}_{ox}^{m}}$ represent the integrated area and the number of the α/α’ anomeric proton signals of CS_ox_ moieties. The obtained results indicated a conjugation degree of 88%, demonstrating that approximately 44% of the aldehyde groups introduced during CS oxidation were successfully converted into imine linkages with MTX, corresponding to 183 ± 12 mg of MTX per g of CS_ox_MTX. The conjugation degree determined by ^1^H-NMR was further validated by UV–Vis spectrophotometric analysis. Comparison of the UV–Vis spectra of MTX and CS_ox_MTX (Supplementary Figure S2), together with quantification based on an MTX calibration curve (λ = 370 nm), revealed a drug loading of 173 ± 9 mg of MTX per g of CS_ox_MTX, in excellent agreement with the value obtained by ^1^H-NMR analysis.

**Figure 3:**
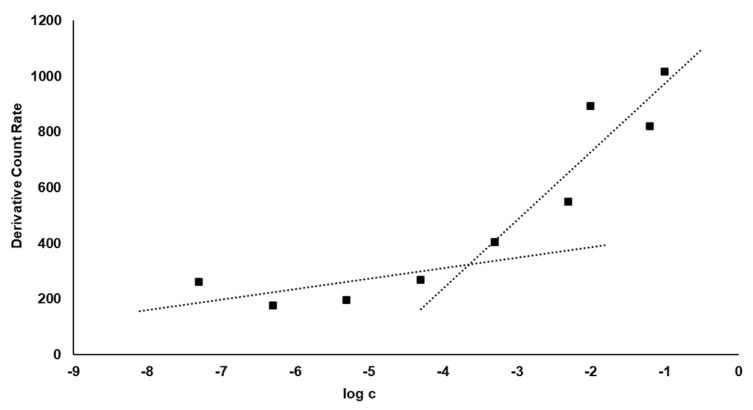
Determination of CAC of CS_ox_MTX by DLS measurements.

The insertion of MTX moieties onto the polysaccharide backbone is expected to modify the hydrophilic to lipophilic balance of the CS_ox_MTX. Therefore, the ability of the prodrug to self-assemble in an aqueous environment, resulting in the formation of nanoparticle structures (CSMXPs), was investigated. In this regard, the critical aggregation concentration (CAC) is an important indicator of micellar stability, as lower CAC values reflect a greater tendency of amphiphilic molecules to form self-assembled structures even at low concentrations. The CAC can be determined by using techniques capable of detecting significant changes in the structural properties of the system below and above this threshold, corresponding to the transition from the monomeric to the micellar state. In this study, the CAC was measured by dynamic light scattering (DLS). In this technique, when coherent monochromatic light passes through a solution containing particles or droplets, a portion of the incident light is scattered according to the optical properties of the system. Micelle formation is evidenced by a marked increase in scattered light intensity occurring with a slight increase in monomer concentration, as micellar aggregates scatter light more efficiently than dissolved species, including free monomers and counterions [36]. The concentration at which this sharp increase occurs is considered equivalent to the CAC value (Figure 3).

The results indicated a CAC of 2.2 × 10^−4^ mg mL^−1^, which is consistent with the use of the resulting self-assembled polymeric prodrug nanoparticles.

### 2.2. Synthesis of Self-Assembled Polymeric Prodrug Nanoparticles and In Vitro Delivery Studies

**Figure 4:**
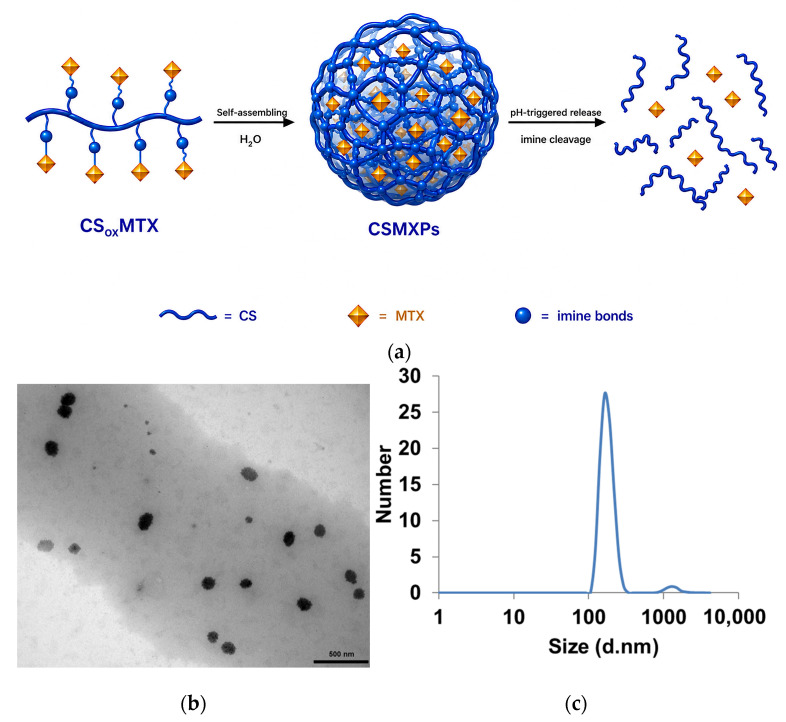
(**a**) Schematic representation of CSMXPs formation by CS_ox_MTX self-assembly and pH-triggered CSMXPs destabilization/MTX release; (**b**) CSMXPs TEM image; (**c**) CSMXPs size distribution by DLS analyses.

Self-assembled polymeric prodrug nanoparticles (CSMXPs) were prepared by dissolving CS_ox_MTX in an aqueous medium followed by the addition of dichloromethane. The organic solvent facilitates solubilization of the hydrophobic components, ensuring the formation of a homogeneous mixture, while sonication provides the mechanical energy required to drive polymer self-assembly and nanoparticle formation (Figure 4a). The resulting nanoparticles were characterized by transmission electron microscopy (TEM) and dynamic light scattering (DLS) (Figure 4b,c).

TEM micrographs revealed a spherical morphology, while the combined TEM and DLS analyses confirmed the presence of a predominant nanoparticle population with a mean diameter of 120 ± 10 nm and a polydispersity index (PDI) of 0.24. A minor secondary population centered at approximately 1000 nm, accounting for 3.2% of the total nanoparticle population, was attributed to the aggregation of CSMXPs. Given the negligible proportion of these aggregates, the sample was considered predominantly monodisperse, with the low PDI value further indicating a relatively narrow size distribution of the main nanoparticle population. These physicochemical characteristics are considered suitable for cancer therapy applications [37]. Furthermore, CSMXPs exhibited a surface charge of −20.4 mV (due to the presence of ionizable groups in both CS_ox_ and MTX moieties) and stability for up to 14 days at room temperature, suggesting good colloidal stability for application as a delivery vehicle.

**Figure 5:**
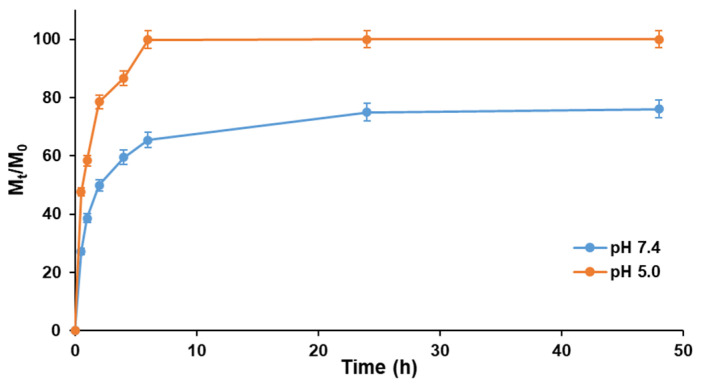
MTX release profile from CSMXPs at pH 7.4 and 5.0.

CSMXPs were engineered to achieve controlled MTX release in response to the acidic intracellular endosomal/lysosomal compartments. Accordingly, the release behavior of MTX was investigated in acetate buffer (pH 5.0), mimicking endosomal/lysosomal acidic conditions, and compared with that observed in phosphate buffer (pH 7.4), representative of physiological conditions (Figure 5).

The release behavior of MTX was governed by the interplay between the drug diffusion through the nanoparticulate matrix and the hydrolytic cleavage of the imine linkage connecting MTX to the polysaccharide carrier. Although CSMXPs exhibited appreciable drug release under physiological conditions, reaching approximately 50% within the first 2 h, MTX liberation was markedly accelerated at acidic pH, where protonation of the imine bond promoted its hydrolysis, resulting in approximately 75% drug release over the same period. The release observed at pH 7.4 can be attributed to the dynamic equilibrium of the reversible imine linkage in aqueous media, together with the high degree of hydration the chondroitin sulfate matrix, which facilitates partial drug diffusion. Furthermore, acidic conditions markedly affected the size distribution of CSMXPs, increasing the mean particle size to 342 ± 95 nm (PDI > 0.6) (Supplementary Figure S3). This behavior suggests that changes in the ionization state of the polymer backbone alter the intermolecular interactions and the hydrophilic–lipophilic balance of the conjugate, thereby promoting nanoparticle aggregation through enhanced intermolecular hydrogen bonding [38]. Therefore, the enhanced release observed at pH 5.0 is likely the result of the combined effects of nanoparticle matrix destabilization and acid-catalyzed hydrolysis of the imine linkage.

To gain further insight into the MTX release mechanism, semi-empirical kinetic models commonly reported in the literature were applied to the experimental data [39]. In particular, the release profiles were analyzed assuming reversible first-order (Equation (3)) and second-order (Equation (4)) kinetics:

(3) $$\frac{M_{t}}{M_{0}}=\;F_{max}\left(1-e^{-{(k}_{R}/F_{max})t}\right)$$

(4) $$\frac{M_{t}}{M_{0}}=\frac{F_{max}(e^{2\left(\frac{k_{R}}{\alpha }\right)t}-1)}{1-2F_{max}+e^{2\left(\frac{k_{R}}{\alpha }\right)t}}$$

In these models, *F_max_* represents the maximum relative release *M_t_*/*M*_0_, while *k_R_* denotes the release rate constant. The affinity of MTX between the carrier and the external release medium was evaluated through the affinity parameter *α*, calculated according to the corresponding model equation. This parameter can be interpreted as the drug partition coefficient between the nanoparticulate matrix and the release medium.

The parameters derived from the fitting of the experimental data using both kinetic models are summarized in Table 1.

The experimental data were accurately described by the reversible first-order model, as indicated by the high correlation coefficients (R^2^ > 0.95), whereas the reversible second-order model provided a poor fit (R^2^ < 0.70) [39]. Moreover, a markedly higher affinity of MTX for the release medium was observed under acidic conditions compared with physiological conditions, as demonstrated by an approximately tenfold increase in the α parameter. A concomitant increase of about 30% in the release rate constant k_R_ was also observed, indicating that acidic pH enhances both the rate and extent of MTX release. These findings are consistent with the acid-catalyzed hydrolysis of the imine linkage, which promotes drug detachment from the carrier and accelerates its release into the surrounding medium.

### 2.3. In Vitro Biological Activity

To investigate the cellular uptake of the nanoprodrug system, CS_ox_ was labeled with fluorescein isothiocyanate (FITC) before MTX conjugation, and the resulting fluorescent nanoparticles (l-CSMXPs) were incubated with human cervical adenocarcinoma HeLa cells. FITC is one of the most widely used fluorescent tracers for labeling biomaterials and biomolecules for imaging applications due to its ability to react with nucleophilic functional groups, including hydroxyl groups [40].

**Figure 6:**
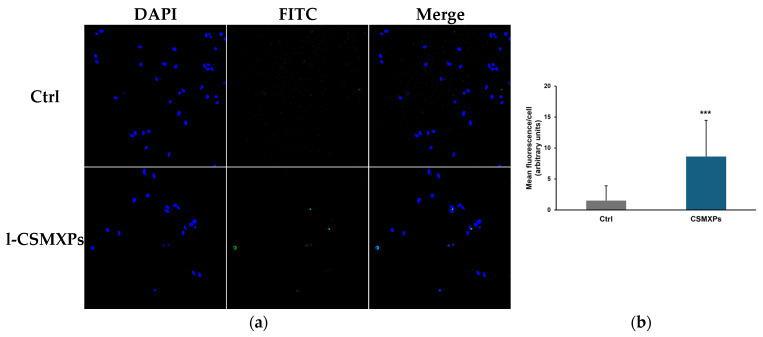
(**a**) Representative confocal microscopy images of HeLa cells after 18 h incubation with medium alone (Ctrl) or FITC-labeled CSMXPs (l-CSMXPs) at 18 μM MTX equivalent concentration. (**b**) Mean fluorescence intensity per cell; *** *p* < 0.001 vs. Ctrl (Student’s *t*-test).

Cell nuclei were counterstained with DAPI, while nanoparticle internalization was assessed by monitoring the FITC fluorescence signal. As shown in Figure 6a, a green fluorescence signal was clearly detected following incubation with l-CSMXPs. The merged images confirmed the intracellular localization of the FITC-labeled nanoprodrug in DAPI-stained cells, demonstrating its efficient cellular uptake. Furthermore, quantitative analysis of the mean fluorescence intensity per cell (Figure 6b) revealed a significant increase in intracellular fluorescence compared with untreated cells. These findings demonstrate that CSMXPs undergo efficient cellular internalization in HeLa cells, supporting their ability to act as intracellular drug delivery vehicles. The observed uptake may be attributed to the nanoscale dimensions of the nanoparticles and to the interaction of the chondroitin sulfate shell with cell-surface receptors, including CD44, scavenger receptors, and proteoglycans, which are known to mediate the recognition and internalization of glycosaminoglycan-based materials. Having established the efficient intracellular uptake of CSMXPs, we next investigated whether this behavior translated into an enhanced antiproliferative effect compared with free MTX.

**Figure 7:**
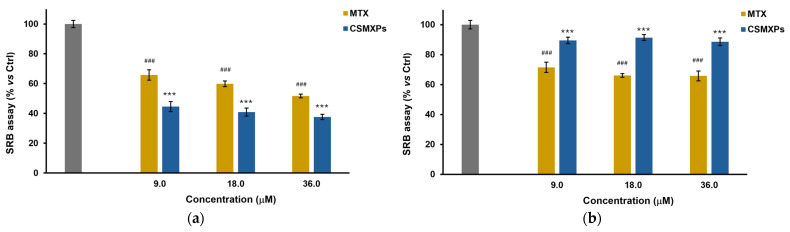
Cell viability of (**a**) HeLa and (**b**) MCF-10A after 72 h treatment with MTX and CSMXPs at different MTX equivalent concentrations. *** *p* < 0.001 vs. MTX; ^###^ *p* < 0.001 vs. Ctrl.

Since oxidized chondroitin sulfate (CS_ox_) alone did not induce significant cytotoxic effect on either HeLa or MCF-10A cells (Supplementary Figure S4), the antiproliferative activity of CSMXPs was evaluated by comparison with free MTX after 72 h of incubation at MTX-equivalent concentrations ranging from 9.0 to 36.0 μM (Figure 7).

As expected, free MTX induced a concentration-dependent reduction in cell viability in both cell lines, highlighting its limited selectivity between cancerous and non-tumorigenic cells. At the highest concentration tested, cell viability decreased to approximately 51% and 65% in HeLa and MCF-10A cells, respectively.

In contrast, the CS-based nanoprodrug exhibited a markedly different biological behavior. In non-tumorigenic MCF-10A cells (Figure 7b), CSMXPs showed minimal cytotoxicity, maintaining cell viability above 88% even at the highest MTX-equivalent concentration tested. Notably, the viability of MCF-10A cells remained consistently high (>88%) across the entire concentration range investigated, indicating that the cytotoxicity of CSMXPs was only slightly influenced by increasing MTX-equivalent concentrations. This behavior suggests that under pH 7.4 conditions, a limited amount of MTX is released, thus significantly preserving the viability of healthy cells.

Conversely, CSMXPs displayed enhanced antiproliferative activity against HeLa cells compared with free MTX (Figure 7a). Cell viability progressively decreased with increasing concentration, reaching approximately 37% at 36 μM, corresponding to a significantly greater inhibition of cancer cell growth than that achieved with the free drug. The opposite trends observed in healthy and cancer cells demonstrate an improved therapeutic selectivity of the nanoprodrug system. This behavior is consistent with the enhanced MTX release observed at pH 5.0, likely resulting from the combined effects of nanoparticle matrix destabilization and acid-catalyzed hydrolysis of the imine linkage. Furthermore, the preferential interaction of CS with tumor-associated cellular receptors and extracellular matrix components may potentially contribute to enhanced interaction of CSMXPs with HeLa cells, further amplifying the antitumor efficacy of the system. Overall, these findings demonstrate that CSMXPs effectively combine reduced toxicity toward healthy cells with enhanced anticancer activity, highlighting their potential as selective nanoprodrugs for cancer therapy.

## 3. Materials and Methods

### 3.1. Synthesis and Characterization of Oxidized Chondroitin Sulfate

Chondroitin sulfate (CS) was oxidized following as previously described [41]. CS (0.25 g) was dissolved in 5.0 mL distilled water, then sodium periodate (NaIO_4_, 0.09 g, 0.42 mmol) was gradually added and the mixture was reacted for 6 h at room temperature under continuous magnetic stirring. The oxidized chondroitin sulfate (CS_ox_) was purified by dialysis (Pur-A-Lyzer^TM^ Mega 1000 Dialysis Kit, Merck/Sigma Aldrich, Darmstadt, Germany) against distilled water at 20 °C for 72 h and finally recovered by freeze-drying (96% yield).

The CS_ox_ oxidation degree was assessed by titration with hydroxylamine hydrochloride (NH_2_OH·HCl) using methyl orange as the indicator [42]. CS_ox_ (0.02 g) was dissolved in 5.0 mL 0.25 mol L^−1^ hydroxylamine hydrochloride solution containing 0.05% (*w*/*v*) methyl orange and incubated in the dark for 2 h. Afterwards, the solution was titrated with a 0.1 mol L^−1^ sodium hydroxide solution until the endpoint was reached.

All chemicals were purchased from Merck/Sigma Aldrich (Darmstadt, Germany).

### 3.2. Synthesis and Characterization of the Methotrexate Prodrug

Methotrexate prodrug (CS_ox_MTX) was synthesized as follows. In the first step, 0.10 g CS_ox_ were dissolved in 4.0 mL of dimethyl sulfoxide (DMSO) and maintained at 40 °C under magnetic stirring. Subsequently, 2.0 mL MTX solution in DMSO (30 mg mL^−1^) was added dropwise followed by the addition of a few drops of acetic acid. The reaction mixture was stirred for 48 h and the resulting conjugate was purified by precipitation in ethanol and collected by centrifugation (8500 rpm, 10 min, Neya 16 R Centrifuge, Remi Elektrotechnik Ltd., Mumbai, India), washed twice with fresh ethanol to remove unbound MTX, and finally dried under vacuum at room temperature (recovery of 82.5%).

The effective conjugation between CS_ox_ and MTX was confirmed by ^1^H NMR spectroscopy (Bruker Avance 300, Bruker Italy, Milan, Italy; 25 °C; DMSO-d_6_/D_2_O, 1:1 *v*/*v*). The amount of conjugated drug was determined by UV-Vis spectroscopy on an Evolution 201 spectrophotometer (Thermo Fisher Scientific, Hillsboro, OR, USA). A CS_ox_MTX solution was prepared in PBS (0.01 M, pH 7.4), and absorbance was recorded at 370 nm, corresponding to the characteristic λ_max_ of MTX.

The critical aggregation concentration (CAC) was calculated by Dynamic Light Scattering (DLS, Zetasizer Nano ZS, Malvern, UK). CS_ox_MTX suspensions at different concentrations were prepared and analyzed in polystyrene cuvettes; data acquisition and processing were carried out using Zetasizer Nano software (Zetasizer family software update V8.02, Malvern, UK). The correlation functions and photon count rate (kcps, 10^3^ counts per second), without laser attenuation, were recorded for each sample [36]. The intensity-weighted size distributions were calculated by the software based on sample parameters.

For confocal microscopy analyses, FITC-labeled CS_ox_MTX was prepared through FITC labeling of CS_ox_, as previously reported [16], followed by MTX conjugation. An amount of 60 mg of CS_ox_ were dissolved in 3 mL NaHCO_3_ solution (pH 8.5) in the presence of 2.0 mL FITC solution (2.4 mg mL^−1^), and the mixture was incubated at room temperature for 12 h. Then, FITC-labeled CS_ox_ was dialyzed against distilled water for 72 h, freeze-dried, and allowed to react with MTX as previously reported to obtain l-CS_ox_MTX.

All chemicals were purchased from Merck/Sigma-Aldrich (Darmstadt, Germany).

### 3.3. Synthesis and Characterization of Self-Assembled Polymeric Prodrug Nanoparticles

Self-assembled polymeric prodrug nanoparticles (CSMXPs) were prepared by dissolving 20 mg CS_ox_MTX in 4.9 mL distilled water. The resulting suspension was magnetically stirred for 12 h, then 0.1 mL dichloromethane was added and the mixture sonicated for 10 min. Subsequently, the organic solvent was removed using a rotary evaporator, and the suspension volume was adjusted to a final volume of 5 mL with distilled water. The same procedure was adopted on l-CS_ox_MTX for the preparation of labeled nanoparticles (l-CSMXPs).

The particle size, PDI and zeta potential of CSMXPs (final concentration 1.0 mg mL^−1^) were analyzed via Dynamic Light Scattering (DLS, Zetasizer Nano ZS, Malvern, UK) at 25.0 ± 0.1 °C, set at 4 mW He-Ne laser operating at a wavelength of 633 nm and scattering angle at 173°. ζ-potential values were calculated by the instrument software, employing the Helmholtz–Smoluchosky equation. The polydispersity index (PDI) was obtained from the instrumental data by inverse Laplace transformation using the Contin algorithm. PDI values ≤ 0.3 were considered indicative of homogeneous and monodisperse nanoparticle populations [43].

Morphological analysis was assessed by transmission electron microscopy (TEM). A drop of the nanoparticle dispersion was placed on a Cu TEM grid (200 mesh, Plano GmbH, Wetzlar, Germany), and the excess sample was removed with a piece of filter paper. A drop of 2% (*w*/*v*) phosphotungstic acid solution was then deposited on the carbon grid for 2 min. The excess of staining agent was removed with filter paper, the sample was air-dried, and the thin film of stained nanoparticles was examined using a Tecnai F30 high-resolution transmission electron microscope (HRTEM) operated at an accelerating voltage of 80 kV (FEI Company, Hillsboro, OR, USA).

All reagents used in this study were purchased from Merck/Sigma-Aldrich (Darmstadt, Germany).

### 3.4. In Vitro Release Experiments

In vitro release experiments were performed under sink conditions by dialysis method. In two different experiments, 2 mL of CSMXPs dispersion were loaded in a dialysis bag (MWCO 12–14 kDa) and dialyzed against 13 mL phosphate (0.01 M, pH 7.4) and acetate (0.01 M, pH 5.0) buffer at 37 °C under continuous magnetic stirring. At pre-established time intervals (0.5, 1, 2, 4, 6 and 24 h), 1.0 mL release medium was withdrawn and replaced with fresh medium. The released drug was quantified by UV–Vis spectrophotometry (Evolution 201 spectrophotometer, Thermo Fisher Scientific, Hillsboro, OR, USA) using a MTX standard calibration curve (10–0.1 μM) prepared under the same conditions.

All chemicals were purchased from Merck/Sigma Aldrich (Darmstadt, Germany).

### 3.5. Cell Culture

Human cervical adenocarcinoma (HeLa; ATCC^®^ CCL-2™, Manassas, VA, USA) and non-tumorigenic breast epithelial (MCF-10A) cell lines were obtained from the American Type Culture Collection (ATCC, Manassas, VA, USA). HeLa cells were cultured in RPMI 1640 supplemented with 10% FBS and 1% penicillin/streptomycin (10,000 units mL^−1^/10,000 µg mL^−1^), whereas MCF-10A cells were maintained in DMEM/F-12 containing 5% FBS, 1% L-glutamine, and 1 mg mL^−1^ penicillin/streptomycin. For regular maintenance, cells were kept at 37 °C under 5% CO_2_ and a humidified atmosphere for up to 20 passages. Experiments were strictly restricted to cells within 30 passages from resuscitation and within 4 months of thawing. Mycoplasma negativity was assessed monthly (MycoAlert, Lonza, Milan, Italy), and cell line authentication was performed quarterly at our Sequencing Core via short tandem repeat analysis (AmpFLSTR Profiler Plus PCR Amplification Kit; Applied Biosystems, Monza, Italy).

### 3.6. Cell Proliferation Assay

The antiproliferative effects of free MTX and CSMXPs were evaluated in both MCF-10A and HeLa cell lines using the Sulforhodamine B (SRB) colorimetric assay. Cells were plated in the appropriate medium (1 × 10^4^/well) in 96-well plates and subsequently exposed to increasing concentrations of MTX and CSMXPs at equivalent MTX concentrations (9–35 µM). After 72 h incubation, cells were fixed with 10% trichloroacetic acid (TCA) for 1 h at 4 °C. Then, SRB was added for staining, and cells were washed 3 times with a 1% acetic acid solution. Absorbance was measured at 540 nm using a microplate reader (Multiskan FC, Thermo Fisher Scientific, Hillsboro, OR, USA) [44].

### 3.7. Cell Uptake Experiments

HeLa cancer cells were seeded at a density of 2.0 × 10^4^ cells/well upon Lab-Tek II Chamber Slide w/Cover (Thermo Fisher Scientific, Hillsboro, OR, USA) and cultured overnight in complete medium. The following day, cells were treated for 18 h with l-CSMXPs at 18 μM MTX equivalent concentration. At the end of treatment, cells were fixed for 15 min at 37 °C using 4% paraformaldehyde and nuclei were counterstained with DAPI (0.2 μg mL^−1^). Fluorescence (FITC excitation at 495 nm and emission and 520 nm) was visualized at 20x magnification using a confocal laser scanning microscope (Fluoview FV300, Olympus, Segrate, Milan, Italy). For each experimental condition, the mean fluorescence intensity per cell was measured in three distinct microscopic fields and used as an indicator of intracellular l-CSMXPs accumulation.

All chemicals were from Merck/Sigma Aldrich (Darmstadt, Germany).

### 3.8. Statistical Analyses

All results are expressed as mean ± standard deviation (SD), based on at least three independent experiments, each performed in triplicate, unless otherwise specified. Statistical significance between two groups was assessed using Student’s *t*-test, whereas comparisons between three or more groups were performed by one-way analysis of variance (ANOVA). A *p*-value ≤ 0.05 was considered statistically significant.

## 4. Conclusions

A pH-responsive chondroitin sulfate–methotrexate polymeric prodrug conjugate was successfully synthesized through imine bond formation between oxidized CS and MTX. The amphiphilic conjugate exhibited a high degree of MTX conjugation and spontaneous self-assembly into stable nanoparticles with physicochemical characteristics suitable for anticancer drug delivery applications. The resulting CSMXPs showed good colloidal stability and a pH-responsive release behavior, with accelerated MTX liberation under tumor-mimicking acidic conditions, likely due to acid-catalyzed hydrolysis of the imine linkage.

Biological studies demonstrated that the nanoprodrug significantly enhanced the differential cytotoxic response between cancerous and non-tumorigenic cells. Furthermore, fluorescence imaging confirmed the efficient uptake of the nanoparticles by cancer cells, supporting the potential of the system for intracellular drug delivery. The combination of potential chondroitin sulfate-mediated cellular interactions, nanoparticle self-assembly, and pH-triggered drug release provides a promising strategy for improving the efficacy and safety of MTX-based chemotherapy.

These encouraging in vitro findings provide a strong foundation for the further development of CS-based nanoprodrugs and support their potential application in future cancer drug delivery strategies. Future studies will focus on in vivo investigations to assess biodistribution, pharmacokinetics, tumor accumulation, therapeutic efficacy, and systemic safety, thereby providing a comprehensive evaluation of the translational potential of the proposed delivery platform.

## Figures and Tables

**Table 1:** Kinetic parameters for MTX release.

Model	Parameter	pH 7.4	pH 5.0
Reversible-first order	R^2^	0.95	0.96
	*F_max_*	0.70	0.96
	*α*	2.3	24
	*k_R_*	0.71	0.92
Reversible-second order	R^2^	<0.70	<0.70

## Data Availability

Data are available from the corresponding author upon reasonable request.

## Supplementary Materials

The following supporting information can be downloaded at https://www.mdpi.com/article/10.3390/molecules31152578/s1, Figure S1. 1H-NMR spectrum of CS in DMSO-d6/D_2_O; Figure S2. UV-Vis spectra of MTX and CS_ox_MTX in PBS 10^−2^ M; Figure S3. CSMXPs size distribution by DLS analyses at pH 5.0; Figure S4. Cell viability of HeLa and MCF-10A after 72 h treatment with CSox at different concentrations.
