# Supplementary material for: Chondroitin Sulfate-Based Self-Assembling Nanoprodrug for Controlled Methotrexate Delivery in Cancer Therapy
Source: Molecules. 2026 Jul 24;31(15):2578. doi: 10.3390/molecules31152578 (PMC13468386; doi:10.3390/molecules31152578)
Supplement: Supplementary file 1 [file molecules-31-02578-s001.zip › molecules-4429085-supplementary.pdf]

Supplementary Materials

Chondroitin Sulfate-Based Self-Assembling Nanoprodrug for Controlled Methotrexate Delivery in Cancer Therapy

Ludovica Scorzafave †, Michele Pellegrino †, Giuseppe Cirillo *, Marco Fiore, Roberta Pino, Diana Amantea, Antonella Leggio, Fiore Pasquale Nicoletta, Francesca Iemma and Manuela Curcio

Department of Pharmacy, Health and Nutritional Sciences, University of Calabria, 87036 Rende (CS), Italy
† These authors contributed equally to this work
* Correspondence: giuseppe.cirillo@unical.it; Tel.: +39 0984493208

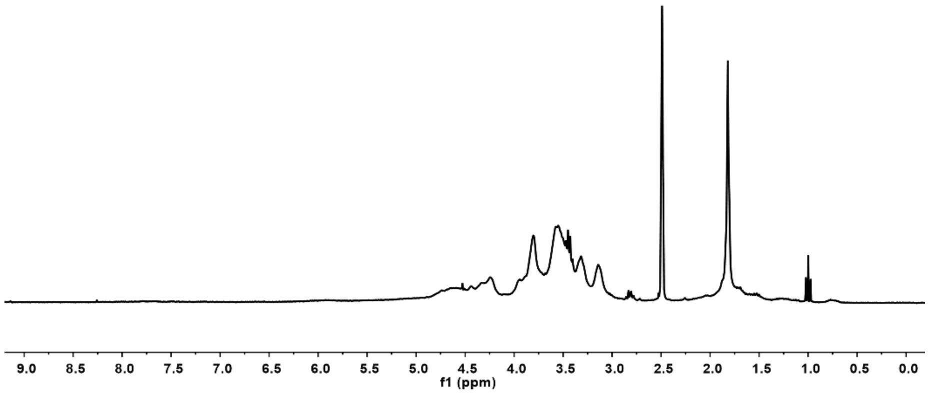

Figure S1. ^{1}H-NMR spectrum of CS in DMSO-d6/D_2O.

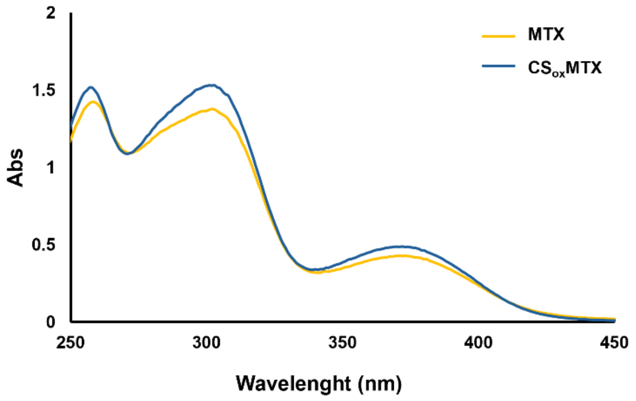

Figure S2. UV-Vis spectra of MTX and CS_{ox}MTX in PBS 10^{-2} M.

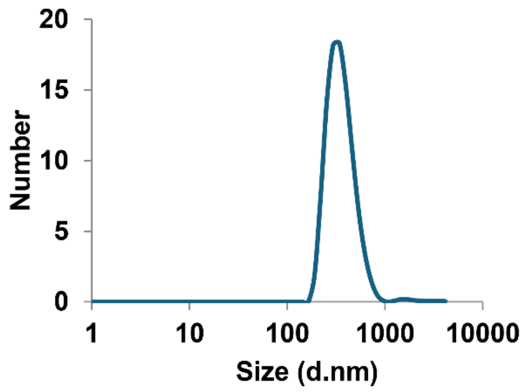

Figure S3. CSMXPs size distribution by DLS analyses at pH 5.0.

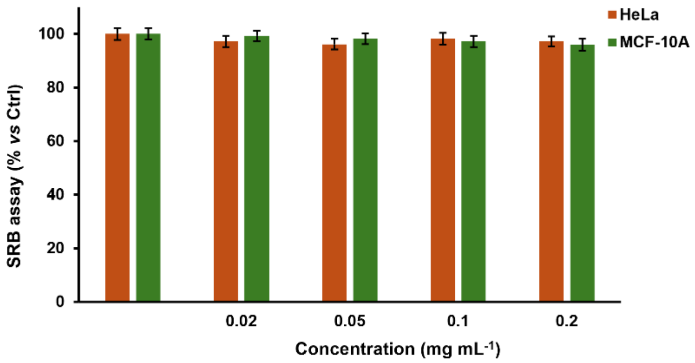

Figure S4. Cell viability of HeLa and MCF-10A after 72 h treatment with CS_{ox} at different concentrations.
